# Selected Aspects of Tobacco-Induced Prothrombotic State, Inflammation and Oxidative Stress: Modeled and Analyzed Using Petri Nets

**DOI:** 10.1007/s12539-018-0310-7

**Published:** 2018-12-24

**Authors:** Kaja Gutowska, Dorota Formanowicz, Piotr Formanowicz

**Affiliations:** 1 Institute of Computing Science, Poznan University of Technology, Piotrowo 2, 60-965 Poznan, Poland; 2 Department of Clinical Biochemistry and Laboratory Medicine, Poznan University of Medical Sciences, Rokietnicka 8, 60-806 Poznan, Poland; 3 Institute of Bioorganic Chemistry, Polish Academy of Sciences, Noskowskiego 12/14, 61-704 Poznan, Poland

**Keywords:** Atherosclerosis, Prothrombotic state, Cigarette smoke, Modeling, Petri nets, t-invariants, 92C42, 93A30, 97M10

## Abstract

Many factors, such as endothelial dysfunction, inflammation and hemostatic disturbances, affect formation and progression of atherosclerotic plaque. In our study, we have focused on hemostatic disturbances with particular emphasis on the extrinsic pathways of coagulation. Thrombin is a main enzyme of coagulation and it is engaged in many different subprocesses. It leads to activation of factors of the coagulation cascade, transition of fibrinogen to fibrin monomer, endothelial damage, inflammation, activation of platelets and proliferation. In our study, selected aspects of disorders in prothrombotic states influenced by cigarette smoke have been modeled and analyzed. Tobacco-induced increased tissue factor, which is associated with less plasminogen activator and increased plasminogen activator-1 inhibitor, has been included in the presented model. These disorders together with accompanying inflammatory state are closely related to thrombus formation and cardiovascular disease promotion. The proposed model has been built using Petri nets and the analysis has been based mainly on t-invariants. Using the Petri net theory to model and analyze the investigated phenomena allows to better understand them by revealing some interesting dependencies in the studied biological system. It explains how tobacco smoke affects the analyzed processes and how harmful these effects are.

**Electronic supplementary material:**

The online version of this article (10.1007/s12539-018-0310-7) contains supplementary material, which is available to authorized users.

## Introduction

Inflammatory process, oxidative stress and hemostasis disturbances play a central role in atherosclerosis development and its clinical consequences [1]. Smoking is a major risk factor for heart disease that aggravates all of the mentioned harmful processes in the arteries wall.

Normally, the endothelial cells of the arterial wall resist leukocyte adhesion and aggregation and promote fibrinolysis [2]. This is the state of endovascular homeostasis. However, if any additional harmful factor appears, the situation is changing and becomes very dynamic. The endothelium responds immediately to an inciting injury to the vascular endothelium, in order to get homeostasis state again. One such factor is tobacco smoke, promoting proinflammatory cytokines expression. In response to it, activated endothelial cells express on their surface adhesion molecules, what mediates an attachment of circulating monocytes and lymphocytes. The atherosclerotic cascade begins. Thus, once the monocytes start to adhere to the activated endothelium, chemokines provide a chemotactic stimulus that induces monocytes to enter the intima of the artery wall [3]. Here they mature into macrophages expressing scavenger receptors that allow the absorption of modified lipoprotein particles. Next, macrophages are transformed into lipid laden foam cells and proliferate within the intima. Here they support and enhance the inflammatory process by releasing proteolytic enzymes (metalloproteinases) that decrease the collagen content in the fibrous cap and the plaque becomes rupture-prone. Finally, the blood that penetrates through the ruptured plaque to its core comes into contact with TF—a major factor activating the extrinsic coagulation system. Under normal conditions, the tissue factor is expressed only in the surrounding layers of the artery wall, which allows for its activation only if the vessel is damaged from the outside. However, in the atherosclerotic plaque infiltrated by macrophages, TF is expressed in the inner endothelial layer of the artery [4]. Inflammation seems to be a key regulator of the fibrous cap fragility, as well as the thrombogenic potential of the plaque. In addition, smoking stimulates excessive production of reactive oxygen species (ROS) and free radicals, which also promote inflammation, LDL oxidation and reduction of nitric oxide (NO) bioavailability (by respiratory burst).

In our study, we have focused on the impact of chronic inflammation and oxidative stress, that are associated with the cigarette smoke, on the selected aspects of atherosclerosis with particular emphasis on the hemostatic system as a crucial modulator of this process.

Hemostasis is a physiological process that stops bleeding at the site of an injury while maintaining normal flow somewhere else in the circulation. This process is tightly regulated. Two main components of hemostasis are distinguished. Primary hemostasis refers to platelet aggregation, adhesiveness and platelet plug formation. Secondary hemostasis involves deposition of insoluble fibrin that is generated by the proteolytic coagulation cascade. There are two interrelated pathways of coagulation, i.e., the contact activation pathway (formerly known as the intrinsic pathway) and the tissue factor pathway (formerly known as the extrinsic pathway). Cigarette smoking has been revealed to increase the ability of coagulation, what finally can lead to obstructive clotting in the cardiovascular system [5–7]. There is no clear statement about the role of the hemostatic system in progression of atherosclerotic lesions [8], however, many experimental data indicate that platelets and coagulation system are prominent factors in the development of atherogenesis and atherothrombosis. Platelets exert a plethora of activities being a link between hemostasis, innate immunity and inflammatory process that underlie atherosclerosis. The local inflammatory process in the artery vessel wall induces a phenotypic switch to proatherogenic endothelium.

The above described process is very complicated and not fully understood. Hence, to gain deeper knowledge about this biological phenomenon system approach should be applied. According to this approach, living organisms as well as their functional blocks as organs, tissues, cells, etc. are complex biological systems. It means that their properties follow not only directly from properties of their basic building blocks but also (or rather mainly) from complex interactions among these blocks. From this follows that for a real deep understanding of the nature of such complicated biological phenomenon it is necessary to analyze them using methods suitable for investigations of complex systems. Many such methods have been developed for years in the area of systems sciences, especially in the context of technical systems. However, a direct application of them to biological systems may be not possible, at least in some cases, since such systems have their specificity or even new methods should be developed. However, in any case, a first step in the systems approach is a construction of a precise model of the investigated biological system. Such a model should be expressed in a language of some mathematical theory. Here, differential equations are used. But in the context of biological phenomena they have some serious limitations. They follow mainly from the fact that in practice usually it is very difficult to obtain precise values of parameters describing quantitative properties of the biological system, which are necessary to build a differential equation-based model. It is one of the reasons for searching for methods of other types. Among them network-based methods, and especially the ones based on Petri net theory, seem to be very promising.

In this paper, we propose a Petri net-based model of the above described biological process. The model was the basis for a formal analysis of the studied phenomenon, whose results are also described.

## Methods

As has been already mentioned, the process comprising the tobacco-induced prothrombotic state leading to the development of atherosclerosis has been modeled and analyzed using Petri nets. A net of this type has a structure of a weighted directed bipartite graph. It means that it is composed of two disjoint sets of vertices, called places and transitions. They can be connected by arcs but only arcs connecting a place with a transition or a transition with a place is possible (i.e., no two places or transitions can be connected). The arcs are labeled with positive integer numbers called weights. When a Petri net is a model of a biological system, places usually represent its passive elementary components such as substrates or products of chemical reactions, while transitions correspond to active elementary components of the system such as chemical reactions or other basic interactions among passive components. Arcs model causal relationships among active and passive components of the system [9, 10].

One of the important general properties of Petri nets is the dynamics. Obviously, graphs are mathematical objects, where the dynamics is not present, but in Petri nets there are elements of one more type, i.e., tokens. They are located in places and represent an amount of a passive system component modeled by a given place. Tokens can flow from one place to another via transitions and such a flow represents a flow of information, substances, etc. through the modeled system [9, 10].

In case of biological systems tokens often represent amounts of substrates and products of chemical reactions or other interactions between molecules or their complexes. The flow of tokens is governed by a transition firing rule, according to which a transition is active if tokens occur in every places directly preceding this transition. The number of tokens is equal to at least the weight of the arc connecting the place with the transition. An active transition can be fired, what means that tokens can flow from its pre-places to its post-places, i.e., places which directly succeed the transition. The number of flowing tokens is equal to the weight of a given arc. A distribution of tokens over all places, called a marking of the net, corresponds to a state of the modeled system [9, 11].

Petri nets have a very intuitive graphical representation, i.e., places are depicted as circles, transitions as rectangles, arcs as arrows, tokens as dots or positive integer numbers located in places and weights as positive integer number associated with arcs (if there are no such a number associated with a given arc it means its weights is equal to one). Such a representation helps in understanding the structure of the modeled system and also its behavior during simulation. However, it is not very well suited for a formal analysis of the net. For this purpose another representation, called an incidence matrix, is usually used. Such matrix *A* is composed of *n* rows corresponding to places and *m* columns which are related to transitions. Entry $a_{ij}$ of the matrix is an integer number being a difference between the numbers of tokens residing in place $p_i$ before and after firing transition $t_j$, i.e., $a_{ij}=w(t_{j},p_{i})-w(p_{i},t_{j})$, where *w*(*a*, *b*) is a weight of one (*a*, *b*).

In case of Petri net-based models of biological systems especially important is an analysis based on t-invariants. Such an invariant is vector $x\in \mathbb {N}^m$ being a solution of equation $A\cdot x=0$. While transitions correspond to some elementary subprocesses occurring in the modeled system, t-invariants are counterparts of subprocesses of higher level, which do not change a state of the system. More precisely, with t-invariant *x* there is an associated set $s(x)=\{t_j: x_j>0, j=1,2,\ldots ,m\}$, called its support, which contains transitions corresponding to positive entries of vector *x*. Firing $x_j$ times every transition $t_j\in s(x)$ returns the system to the state in which it was before firing any transition from *s*(*x*) [12–14].

t-invariants which are similar to each other correspond to sets of transitions (i.e., their supports) which have non-empty intersections. From this follows that such invariants are counterparts of subprocesses which are composed of, among others, some common elementary subprocesses (these elementary subprocesses correspond to transitions being elements of the intersections of the supports). Hence, the subprocesses can interact with each other through these common elementary subprocesses. It means that searching for similarities among t-invariants may lead to discoveries of some unknown properties of the system, which follow from the above mentioned interactions. Looking for the similarities can be difficult if there is a great number of t-invariants, what happens very often. In such a case t-invariants can be grouped into sets called t-clusters, where every such a set contains those t-invariants which are similar to each other according to some measure of similarity [12, 15]. t-clusters can be determined using standard clustering algorithms. Grouping t-invariants into t-clusters usually is not sufficient for finding significant similarities between subprocesses but it suggests where they should be looked for (i.e., within the obtained t-clusters).

There are many clustering algorithms and similarity measures and to obtain a meaningful clustering a proper algorithm as well as a similarity measure should be determined. Moreover, a number of clusters should be also carefully chosen. In general, it is not a simple task since there are no general rules for selecting the proper algorithm, the similarity measure and the number of clusters. It should be noted that t-clusters usually correspond to some biological modules of the modeled system whose functions should be determined during the analysis.

Despite that t-invariants can be grouped into t-clusters also transitions can be grouped into the so-called Maximal Common Transition sets (MCT sets) [11]. A set of this type contains transitions which are elements of supports of exactly the same t-invariants. MCT sets partition the set of transitions into disjoint subsets and each of them correspond to some functional module of the analyzed biological system [13, 14].

## Biological Description of Mechanisms Described by the Model

### The Tissue Factor Pathway (the Extrinsic Pathway of Coagulation)

Endothelial dysfunction, inflammation, oxidative stress and disorders in prothrombotic states are crucial for atherosclerosis development and its clinical consequences.

**Fig. 1:**
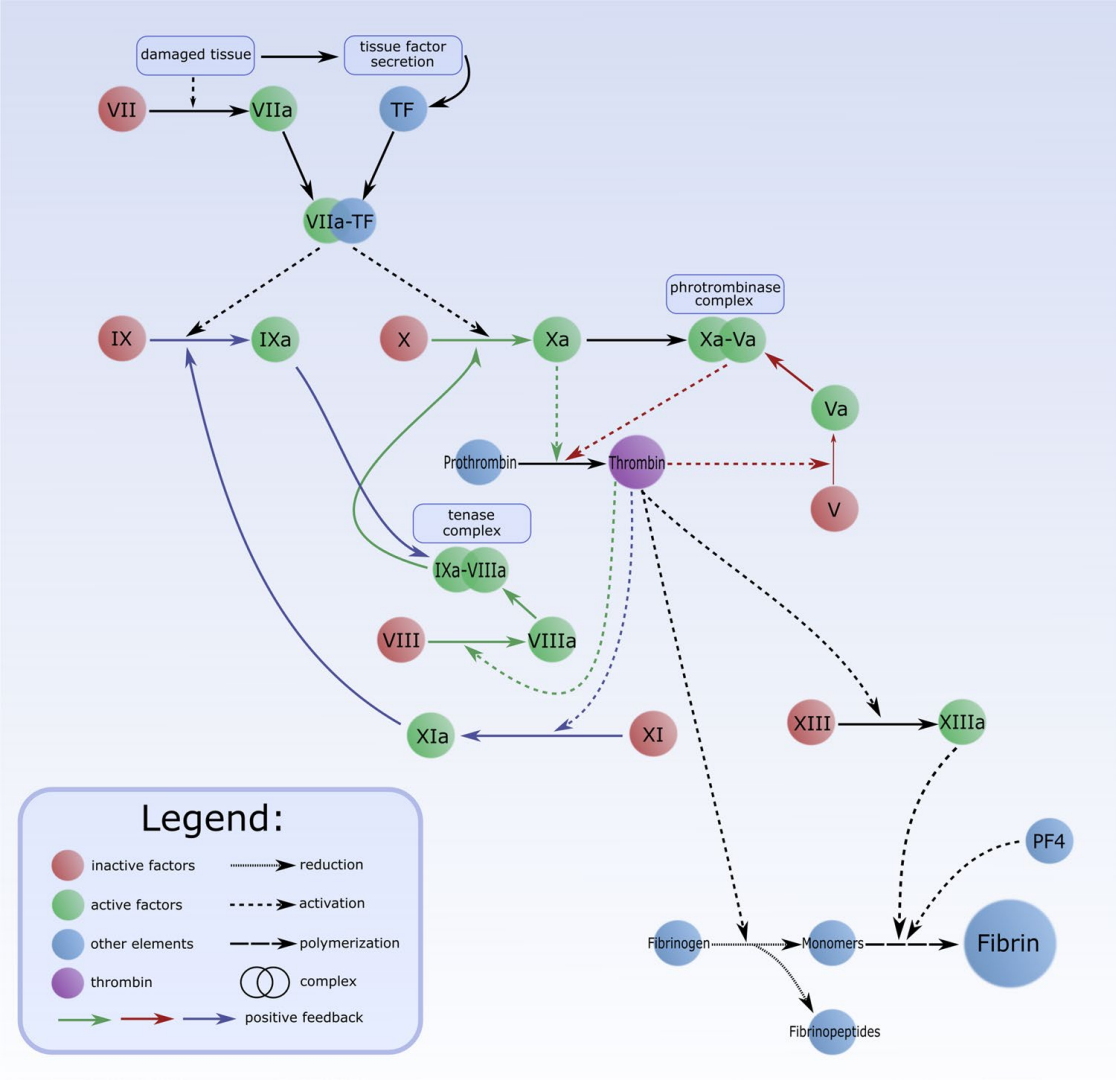
A scheme of the extrinsic pathways of coagulation. Colors of arrows (green, red and blue) correspond to three pathways of positive feedback of thrombin formation

In the proposed Petri net-based model, selected aspects of the coagulation, with particular emphasis on the TF pathway of coagulation (the extrinsic pathway), have been taken into account. A scheme of this process is shown in Fig. 1. The contact activation pathway (the intrinsic pathway) has been omitted in the model because it is not crucial for the studied phenomenon [16].

Coagulation is a dynamic process and represents a balance between clot formation and the mechanisms that inhibit it (beyond the injury site) [17]. This delicate balance is interrupted whenever the anticoagulant activity of naturally occurring inhibitors is decreased or the activity of the coagulation factors is increased.

The procoagulant pathway starts with tissue damage, leading to TF secretion and thereby to the conversion of factor VII to VIIa. A majority of clotting factors are precursors of proteolytic enzymes known as zymogens that circulate in an inactive form. The activation of each zymogen is depicted by suffixing letter “a”. TF binds with factor VIIa and calcium (VIIa-TF complex), which is necessary for the activation of both factor IX to IXa and X to Xa. As a result activated factor X (Xa) leads to transformation of prothrombin to thrombin. Thrombin is one of the important enzymes that acts on a variety of enzymatic activities, thereby regulating the procoagulantanticoagulant balance. It can activate a few different factors: XIII, XI, VIII, V [16].

Activated factor XIII (XIIIa) is engaged in fibrin polymerization. The extrinsic pathways of coagulation includes positive feedback of thrombin formation. To be precise three pathways of it can be distinguished [16]:

- The first pathway:
  Thrombin leads to activation of factor V to Va. Activated factor V (Va) can form prothrombinase complex with Xa, which leads to prothrombin to thrombin transformation.
- The second pathway:
  Thrombin leads to activation of VIII to VIIIa. Activated factor VIII (VIIIa) with factor IXa can form tenase complex. This complex is engaged in activation of factor X to Xa, which results in prothrombin to thrombin transformation.
- The third pathway:
  Thrombin leads to the activation of factor XI to XIa, what activates factor IX. Activated factor IX (IXa) is a part of tenase complex. This complex is engaged in activation of factor X to Xa, which results in prothrombin to thrombin transformation. It can be noticed that tenase complex is common to two pathways: the second and the third one.

The extrinsic pathways of coagulation is shown in Fig. 1.

### Selected Aspects of Cigarette Smoking

Cigarette smoke is one of the contributing risk factors for the development of cardiovascular diseases. Additionally, smoking stimulates many mechanisms associated with atherosclerosis and thereby it promotes disorders in prothrombotic states. In the proposed model, selected aspects of cigarette smoking has been included, e.g., secretion and induction of different particles playing an important role in many harmful processes. Metals, free radicals and ROS stimulate LDL oxidation. Smoking leads to impaired endothelial prostacyclin production and enhanced platelet–vessel wall interactions [18]. Moreover, imbalanced levels of prostacyclin and thromboxane A2 result in vasoconstriction and platelet aggregation, increasing the risk for cardiac events [19]. Nicotine induces catecholamines, which lead to a decreased quantity of dioxygen ($\hbox {O}_{2}$) and increased quantity of carbon monoxide (CO). Cigarette smoke leads to development of inflammatory environment via increased quantity of neutrophils, lymphocytes and macrophages. Additionally, smoking-mediated increase in polycyclic aromatic hydrocarbons (PAHs) in human organism results in induction of chemokines secretion, this in turn aggravates inflammation. Modification of lipid profile caused by smoking leads to increased quantity of LDL and triglycerides (which can be engaged in endothelial damage) and decreased quantity of HDL, tetrahydrobiopterin ($\hbox {BH}_{4}$) and others. Decreased quantity of $\hbox {BH}_{4}$ results in limited NO synthesis. This study focused on the selected aspects of prothrombotic states, which are additionally stimulated by cigarette smoking. To be more precise, cigarette smoking leads to an increased quantity of TF, an inhibition of plasminogen activator and an increased quantity of PAI-1. Other mechanisms included in the model, e.g., initiation and further development of endothelial dysfunction and the role of NO, have been described in [20].

## The Model

### The Modeled System

**Fig. 2:**
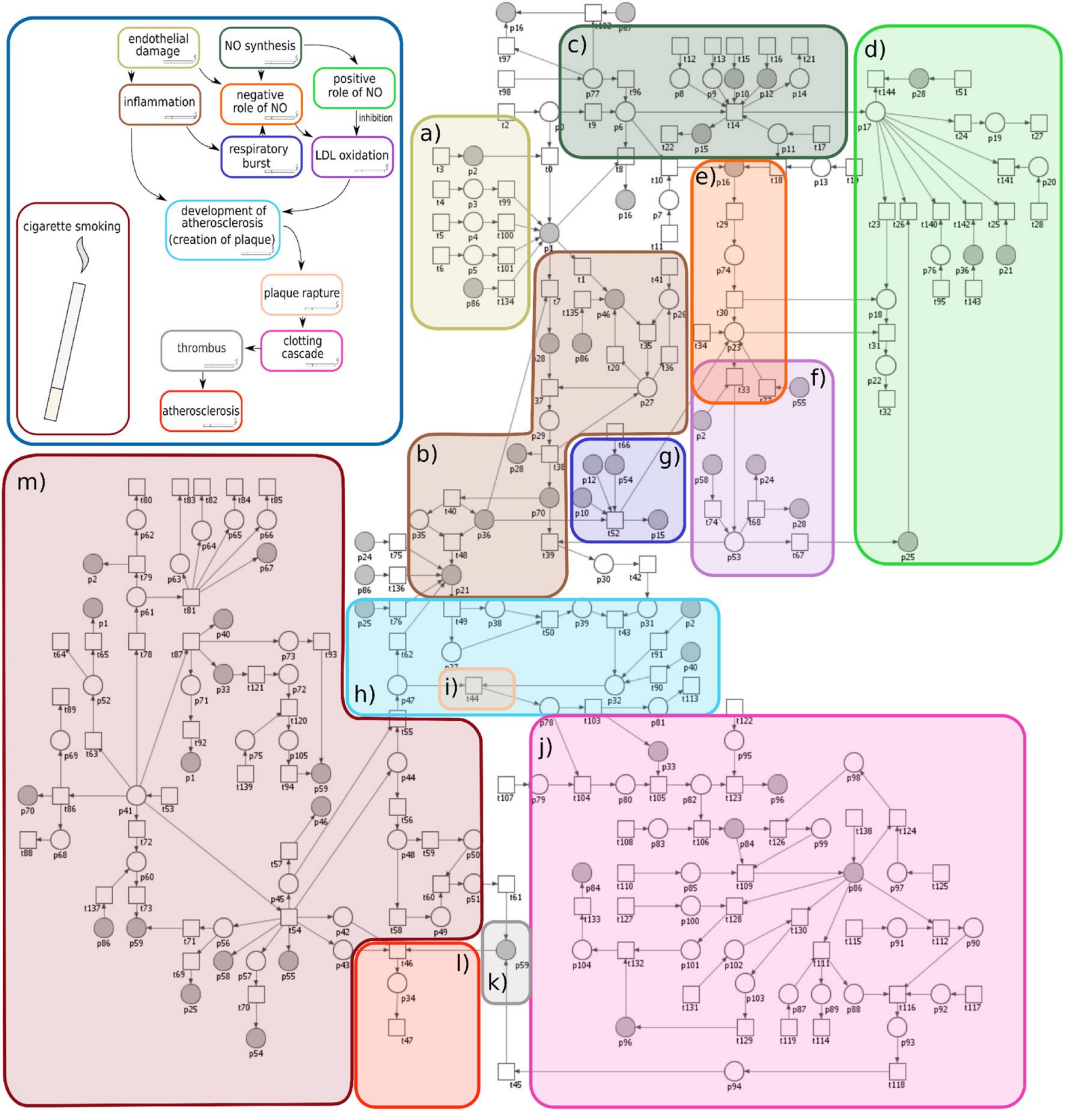
The proposed model has been divided into 13 blocks. The colors of the 13 blocks correspond to the colors in the block diagram located in the top left corner. This block diagram presents the main mechanisms and biological components, which are included in the model. The block called “cigarette smoking” has no arrows, but for better readability a small icon of a cigarette occurs in other blocks. It symbolized the influence of a cigarette smoke on a particular processes

The proposed Petri net model has been built using open-source software called Snoopy [21] and analyzed using free (licensed under the Artistic License 2.0), stand-alone Java application called Holmes [22]. The model is shown in Fig. 2. The block diagram located in the top left corner of this Figure includes the main elements and mechanisms contained in the proposed Petri net-based model. In almost all blocks, a small cigarette has been drawn, what symbolizes the effect of a cigarette smoke on a particular processes. This scheme illustrates mechanisms that are additionally stimulated by a cigarette smoke. Moreover, Fig. 2 includes the Petri net based model divided into 13 blocks. For better transparency of the model different colors of frames (which correspond to mechanisms and biological elements) has been used. The fact that the frames in the diagram located in the top left corner and in the Petri net based model have the same colors is important, because each color corresponds to some biological process or components. Processes which have been distinguished in the Petri net model are: (a) endothelial dysfunction caused by different factors (e.g., high LDL level, high blood pressure, toxins, thrombin), (b) inflammatory response of endothelial damage (which stimulates chemokines and monocytes adhesion), (c) NO synthesis, (d) positive role of NO (when quantity of NO is sufficient for correct the functioning and inhibition of harmful processes), (e) negative role of NO (when quantity of NO is not sufficient for correcting the functioning and stimulates LDL oxidation), (f) LDL oxidation, (g) respiratory burst, (h) development of atherosclerosis (formation of plaque), (i) plaque rapture, (j) coagulation (clotting cascade), (k) formation of thrombus, (l) atherosclerosis, (m) cigarette smoking.

Cigarette smoke is directly or indirectly correlated with almost all of these processes and additionally stimulates harmful mechanisms, what is shown in the diagram located in the top left corner. The names of places and transitions of the proposed model are included in Supplementary Materials. Some rows in Table S1 in Supplementary Materials correspond to facilitation, e.g., $$\hbox {p}_{67}$$ less quantity of $$\hbox {BH}_{4}$$ (which is caused by cigarette smoke), $$\hbox {p}_{77}$$$$\hbox {BH}_{4}$$ co-factor of eNOS (in normal condition). In this case, two places associated with $$\hbox {BH}_{4}$$ correspond to two different situations. Additionally, Table S2 in Supplementary Materials includes “auxiliary transitions”, there are three types of auxiliary transitions: input, output and internal ones. The input transitions correspond to mechanisms engaged in a production of biological or chemical components. The output transitions correspond to mechanisms engaged in different processes, which are not included in the model, because they are not important for the modeled disease. In biological context input and output “auxiliary transitions” correspond to additional sources for some biological or chemical components. Moreover, the third type of “auxiliary transition” called internal transition is necessary to modeling reversible reactions.

The summary description of subprocesses included in the model (according to Fig. 2):

**Block a)***Endothelial dysfunction*: Endothelial dysfunction is an early marker for atherosclerosis. Many of the risk factors, which lead to atherosclerosis can also cause endothelial dysfunction. A damage of endothelium is one of the major causes of atherosclerotic plaque formation. Initiation of endothelial dysfunction is caused by various factors. This dysfunction is commonly associated with reduced NO bioavailability and is additionally stimulated by cigarette smoke. The damaged endothelium induces expression of chemokines, which allow for monocytes adhesion. Endothelial dysfunction is associated with inflammation response [23–27].

**Block b)***Inflammation (as a response of endothelial damage)*: Endothelium and inflammation play central role in all phases of the atherosclerotic development. Moreover, proinflammatory cytokines and adhesion molecules are engaged in the attachment of monocytes to the endothelial wall—this mechanism appears to be critical. Movement of monocytes out of the vessel wall (diapedesis) and transformation into macrophages is typical inflammatory response. The main purpose of the macrophages is to get rid of damaged cells. Cigarette smoke is an additional source of cytokines and macrophages, which stimulates inflammation [2, 3, 25, 27, 28].

**Block c)***NO synthesis*: NO is known as a crucial modulator of vascular disease and it is produced by nitric oxide synthases (in endothelial cells NO is generated by eNOS). NO plays important functional roles in various of physiological systems, including cardiovascular diseases (CVDs). Moreover, nitric oxide can play a dual role which depends on its concentration (positive and negative roles) [23, 25, 29, 30].

**Block d)***Positive role of NO*: When biochemical and molecular mechanisms which regulate bioavailability acts correctly, then a quantity of NO is sufficient for proper functioning. NO regulates blood pressure, platelets aggregation, prevents smooth muscle cell proliferation and leukocyte adhesion. Moreover, nitric oxide inhibits LDL oxidation and opposes the effects of endothelium-derived vasoconstrictors [23, 25, 29–31].

**Block e)***Negative role of NO*: When biochemical and molecular mechanisms lead to a decrease of NO bioavailability, then a quantity of NO is insufficient for proper functioning. Impairment of NO bioavailability favor CVDs and leads to events or actions that promote atherosclerosis, such as vasoconstriction, platelet aggregation, proliferation of smooth muscle cell, leukocyte adhesion, and oxidative stress [19, 23, 25, 32, 33].

**Block f)***LDL oxidation*: A crucial event in the early stages of atherosclerosis development is accumulation of foam cells (built with oxidized LDL form) derived from macrophages. Moreover, nitric oxide plays important role in LDL oxidation through reaction of NO with superoxide anion radical. This reaction has the dual effect, i.e., it leads to a reduction of NO bioavailability, but also to a production of the potent oxidant peroxynitrite (this mechanism prevents LDL oxidation) [23, 25, 32, 34].

**Block g)***Respiratory burst*: A respiratory burst is a process by which phagocytic cells consume large amounts of oxygen during phagocytosis, mainly via activation of NADPH oxidase and release of free radicals. To be precise, respiratory burst leads to a production of superoxide anion radical, which is involved in LDL oxidation. Cigarette smoking additionally stimulates respiratory burst and leads to decreasing of NO bioavailability [19, 25, 33].

**Block h)***Development of atherosclerosis (formation of plaque)*: There exist several phases associated with a development of atherosclerotic plaque, i.e., endothelial dysfunction, inflammation, reparative phase and thrombotic phase. In case of atherosclerosis development LDL level is high, which results in transformation of macrophages into foam cells as a consequence of an uptake of oxidized LDL form (oxLDL). Foam cells are a crucial component of atherosclerotic plaque [3, 16, 25, 32, 35, 36].

**Block i)***Plaque rupture*: Atherosclerotic plaque is associated with accumulation of cholesterol and other lipid compositions in vascular wall (this deposit is covered by fibrous cap). Atherosclerotic plaque may break off, which results in a production of blood clots and it can cause a thrombus. In other words, the foam cells die leading to release of necrotic core, which is built with oxidized LDL firm and which is highly thrombotic [25, 32, 37, 38].

**Block j)***Coagulation (clotting cascade)*: A coagulation is a dynamic process, which represents a balance between clot formation and the mechanisms that inhibit it. A decrease of a procoagulant activity of naturally occurring inhibitors or an increase of an activity of the coagulation factors may interrupt this delicate balance [16, 17, 38, 39].

**Block k)***Formation of thrombus*: The tissue factor is the principal initiator of the coagulation cascade. Moreover, an exposure of collagen in the arterial intima activate blood platelets to a formation of thrombus. Additionally, cigarette smoke promotes disturbances in prothrombotic states. These disorders together with accompanying inflammatory state are closely related with thrombus formation and cardiovascular disease promotion. Mechanisms of thrombus formation lead to a progression of narrowing the arterial lumen via rapidly slow-down of a blood flow [16, 25, 35, 37, 38].

**Block l)***Atherosclerosis*: Atherosclerosis as one of the cardiovascular diseases is the major cause of morbidities and mortalities worldwide. Atherosclerosis is a chronic progressive disorder characterized by vascular sclerosis and lumen stenosis, which is due to lipid accumulation, proliferation of vascular smooth muscle cells (VSMC), and platelet aggregation. These mentioned mechanisms are triggered by lipid oxidation, endothelial dysfunction, and inflammation. Moreover, the knowledge about this multifocal immunoinflammatory disease is still incomplete [3, 16, 25, 32, 35, 36].

**Block m)***Cigarette smoking*: The cigarette smoke is one of the contributing risk factors for the development of CVDs. Moreover, smoking additionally stimulates many mechanisms associated with atherosclerosis and thereby it promotes disorders in prothrombotic states. The model presented in paper [20] is focused on the effect of the cigarette smoke on, inter alia, endothelial dysfunction, inflammation, NO bioavailability and LDL oxidation. However, the proposed model in this paper is concentrated on other selected aspects of smoking, inter alia, the influence of cigarette smoke on a pathway of coagulation (clotting cascade). To be more precise, the model includes the following subprocesses: increasing the quantity of TF, inhibition of plasminogen activator, increasing the quantity of PAI-1 [19, 25, 33, 40, 41].

### Inhibition Reaction in the Model

**Fig. 3:**
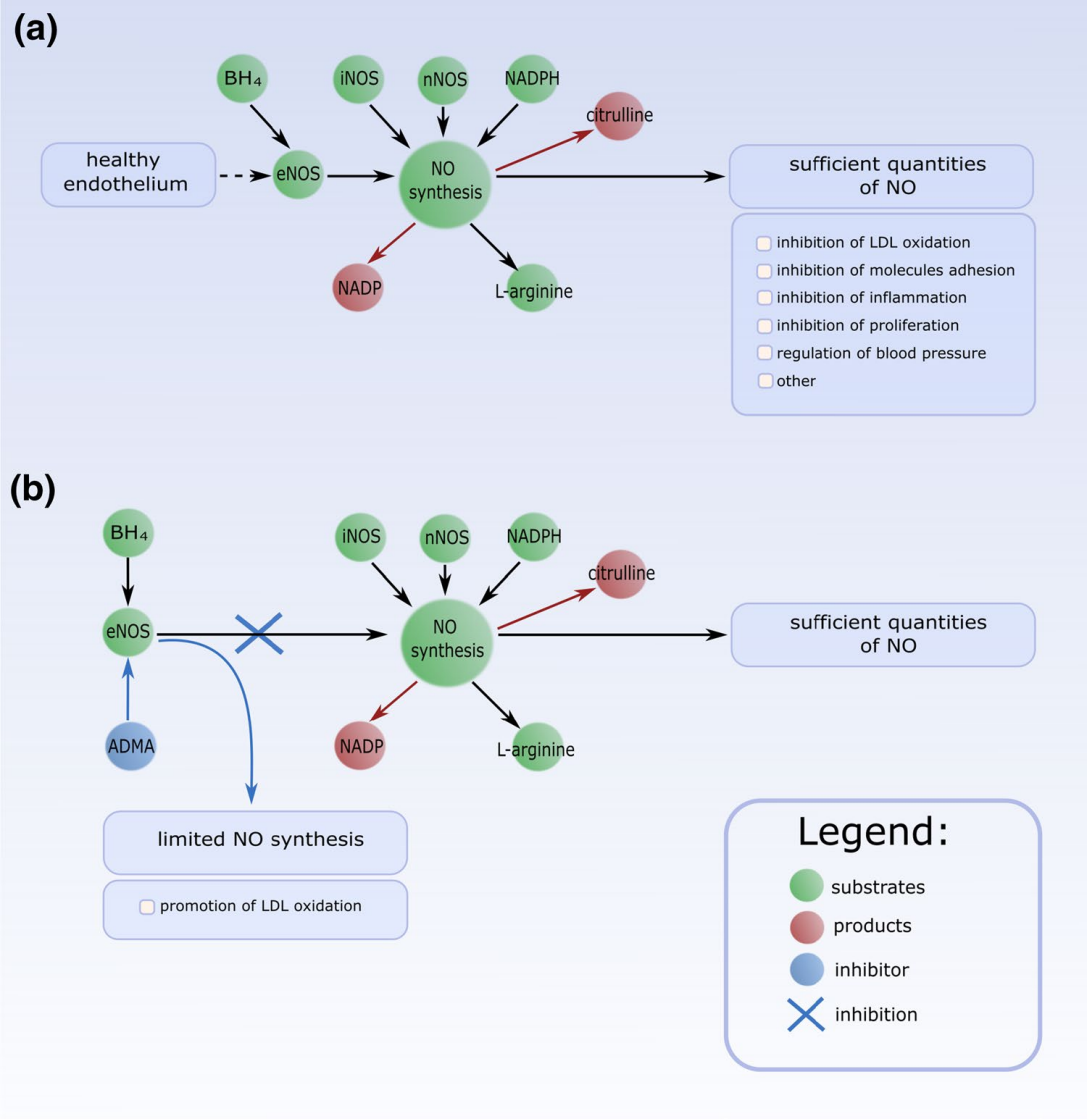
The schema of inhibition reaction, where green circles correspond to substrates, red circles correspond to products, blue circle corresponds to inhibitor and blue arrows correspond to inhibition reaction. **a** It is shown a situation, where NO synthesis is correct and its quantity is sufficient to prevent harmful processes, while in **b** it is shown NO synthesis limited by inhibitor eNOS (ADMA), therefore this process leads to promotion of LDL oxidation

An example of inhibition reaction is shown in Fig. 3. In this schema, two situations are presented. Figure 3a shows the first situation, when the synthesis of NO is proper (all necessary components are present) and the quantity of NO is sufficient to prevent harmful processes. NO regulates blood pressure and can lead to the inhibition of LDL oxidation, molecules adhesion, inflammation, proliferation and other harmful processes, which can be nearly correlated with the development of atherosclerosis. Figure 3b shows the second situation, when NO synthesis is limited by eNOS inhibitor (ADMA) and leads to the promotion of LDL oxidation.

In the presented Petri net-based model various inhibitors and inhibition reactions have been modeled, some of them are:

- L-NMMA (inhibitor of l-arginine)—inhibition of l-arginine leads to limited NO synthesis.
- $\hbox {BH}_{4}$ (eNOS co-factor)—decreased quantity of $\hbox {BH}_{4}$ (caused by the cigarette smoke) leads to a limited NO synthesis
- TF—increased quantity of TF (caused by the cigarette smoke) leads to an inhibition of plasminogen activator, which results in a promotion of prothrombotic states and a creation of thrombus.

**Fig. 4:**
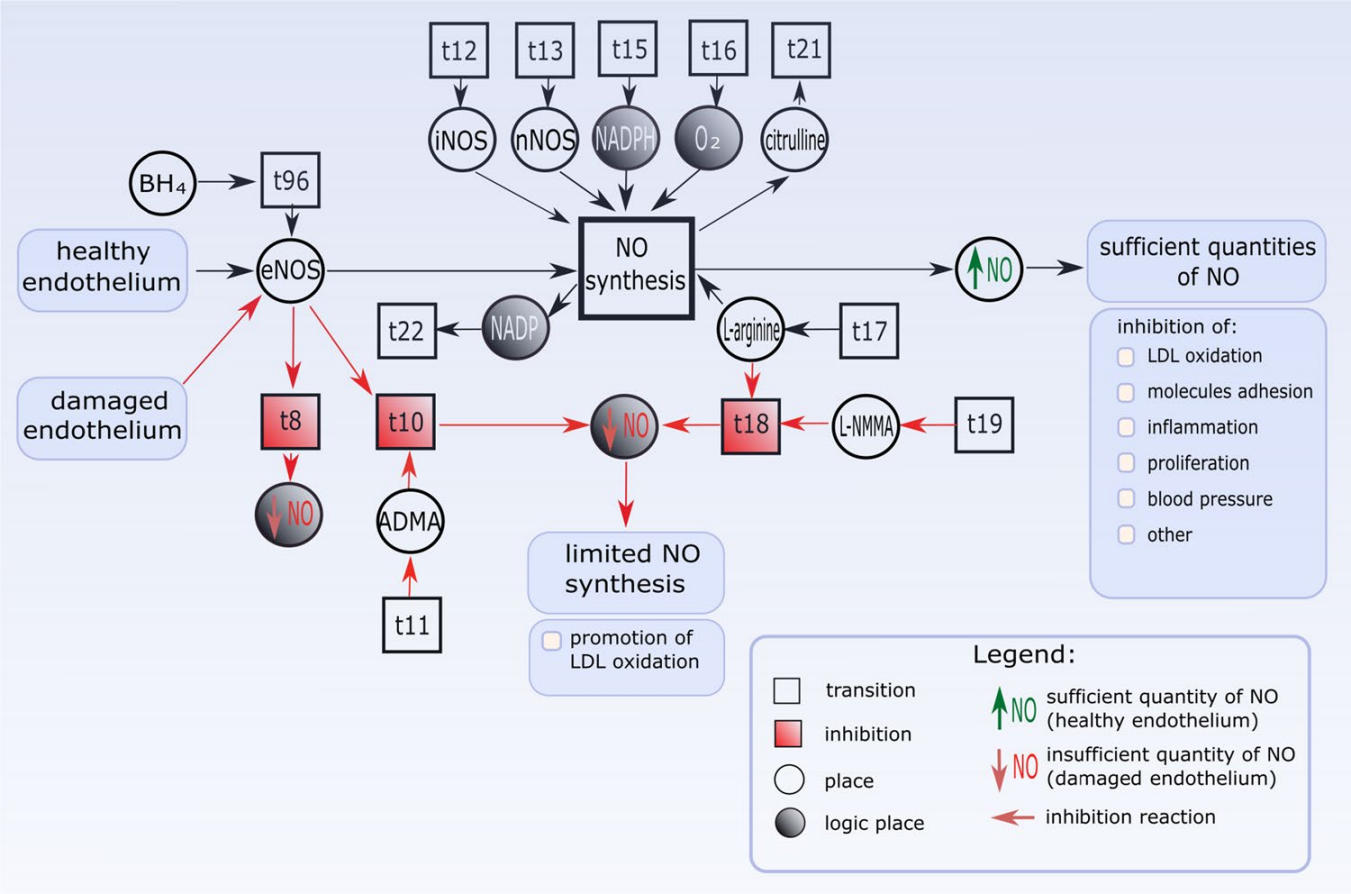
A part of the Petri net-based model that contains two opposite processes: the inhibition of NO synthesis and the proper NO synthesis (presented in Fig. 3). This fragment contains selected factors which have influence on the limited NO synthesis (L-NMMA, ADMA, damaged endothelium). All elements which are associated with the inhibition are marked in red color: $\hbox {t}_{8}$—inhibition of eNOS caused by damaged endothelium, $\hbox {t}_{10}$—inhibition of eNOS caused by ADMA, $\hbox {t}_{18}$—inhibition of l-arginine caused by L-NMMA. These processes lead to an insufficient quantity of NO (limited NO synthesis). Other transitions, i.e., $\hbox {t}_{11}$, $\hbox {t}_{12}$, $\hbox {t}_{13}$, $\hbox {t}_{15}$, $\hbox {t}_{16}$, $\hbox {t}_{17}$, $\hbox {t}_{19}$, $\hbox {t}_{21}$, $\hbox {t}_{22}$ are auxiliary transitions (input or output ones). Transition $\hbox {t}_{96}$ corresponds to increasing the affinity of eNOS to l-arginine

The schemata of both situations (reactions of inhibition and synthesis) that are presented in Fig. 3 are included in the proposed model. The part of the Petri net-based model that covers these two phenomena is shown in Fig. 4. The proposed model has been built using Snoopy software [21], which allows to use inhibitor arcs. But this kind of arc is an extension of classical Petri nets and they are not represented in an incidence matrix. This means that standard analysis methods based on t-invariants (or more generally, methods based on the incidence matrix) cannot be directly used in the case of the nets containing inhibitor arcs. This limitation was a motivation for looking for a method of modeling inhibition reactions using classical Petri net without such arcs. This approach is shown in Fig. 4. In the healthy condition, all components are present, what results in proper NO synthesis. To be more precise, transition “NO synthesis” can be fired when tokens are in places: “eNOS”, “iNOS”, “nNOS”, “NADPH”, “$\hbox {O}_{2}$”, “l-arginine”, and then fired transition “NO synthesis” enables a flow of tokens to place “sufficient quantity of NO”. On the other hand, NO synthesis can be inhibited by different factors, for example, an inhibition of l-arginine caused by a presence of L-NMMA leads to the inhibition of NO. Transition “inhibition of l-arginine caused by L-NMMA” ($\hbox {t}_{18}$) can be fired when tokens are in places “L-NMMA” and “l-arginine”. This action takes token from place “l-arginine”, which prevents correct NO synthesis (because l-arginine is necessary to this synthesis). Moreover, a correctness of the proposed model of inhibition reactions has been confirmed by the results of the analysis of t-invariants.

## Analysis

### Analysis of MCT Sets

The proposed Petri net-based model includes 106 places, 145 transitions and is covered by 2090 t-invariants, what means that every transition is an element of a support of at least one t-invariant. The number of t-invariants is dependent on the nature of the modeled process. Similar t-invariants has been grouped into larger structures, i.e., MCT sets and t-clusters. The model contains 31 non-trivial (i.e., composed of more than one transition) MCT sets and 19 t-clusters. The analysis and biological interpretation for all of the MCT sets is described in Table 1 (which is included in Appendix 1).

### Analysis of Clusters

The analysis of the proposed model is based on t-invariants, which correspond to subprocesses occurring in the modeled biological system. The proposed Petri net includes 2090 t-invariants. Due to the fact that Petri net is covered by large number of t-invariants they are grouped into t-clusters. For the clustering, 7 methods and 8 distance measures have been used. The used algorithms were: average linkage, centroid method, complete linkage, McQuitty’s method, median method, single linkage, and Ward’s method. The used measures were: binary distance, Euclidean distance, Manhattan distance, Canberra distance, maximum distance, Minkowski distance, centered and uncentered Pearson. To identify the best clustering, the Mean Split Silhouette (MSS) index has been used (cf. [13]). MSS evaluates a fit of each t-invariant to its cluster and an average quality of a given clustering [42]. To find the best number of clusters, Calinski–Harabasz (C–H) coefficient has been used (the optimal number of clusters is indicated by the highest value of C–H coefficient) [43]. This coefficient has been calculated for the range from 2 to 20 number of clusters. For the biological analysis a clustering containing 19 t-clusters has been chosen. The clustering has been obtained using Pearson distance measure and average linkage method (UPGMA). The MSS value for this clustering is equal to 0.3988 and C–H has a value of 55.

t-invariants being elements of the same t-cluster can be similar and the corresponding biological subprocesses can influence each other. Moreover, t-clusters correspond to some subprocesses occurring in the analyzed system. For this reason biological interpretation should be assigned to each such a cluster. The analysis of t-clusters can confirm some hypotheses and can lead to discoveries of unknown properties of the modeled system. Biological interpretation of t-clusters is provided in Table 2 (which is included in Appendix 2).

## Discussion

On the basis of the obtained results we have found that endothelial dysfunction, inflammatory response (induction of chemokines and adhesion of monocytes, transformation monocytes to macrophages), and oxidative stress that are caused by various factors lead to limited NO synthesis and may promote LDL oxidation. This part of the model is described in a greater detail in [20].

Cigarette smoke additionally stimulates almost all of the paths in the model. Endothelial dysfunction is caused by modification of lipid profile (which results in increased quantity of LDL) and remodeling of tissue (impairment of FMD). Inflammation is stimulated by increased quantity of macrophages and in consequence by increased quantity of cytokines, which promote LDL oxidation and leads to limited NO synthesis. Moreover, cigarette smoke promotes disturbances in prothrombotic states, which result in thrombus formation and lead to development of atherosclerosis. The effect of cigarette smoking on development of atherosclerosis has been described more precisely in [20].

t-clusters, i.e., $\hbox {c}_{12}$ and $\hbox {c}_{19}$ in Table 2 described biological meaning of correct NO synthesis, what means that quantity of NO is sufficient for proper functioning. t-clusters, i.e., $\hbox {c}_{1}$, $\hbox {c}_{4}$, $\hbox {c}_{5}$, $\hbox {c}_{9}$, $\hbox {c}_{11}$, $\hbox {c}_{17}$ and $\hbox {c}_{18}$ (see Table 2) described biological meaning of limited NO synthesis. The presence of l-arginine inhibitor (L-NMMA) leads to limited NO synthesis ($\hbox {c}_{1}$). Inhibition of $\hbox {BH}_{4}$ leads to inhibition of eNOS and to limited NO synthesis ($\hbox {c}_{4}$). The same situation is caused by cigarette smoke, which results in decreased quantity of $\hbox {BH}_{4}$ ($\hbox {c}_{5}$). Inhibition of eNOS is caused by ADMA ($\hbox {c}_{9}$) and by endothelial dysfunction ($\hbox {c}_{11}$, $\hbox {c}_{17}$, $\hbox {c}_{18}$). In the case of eNOS inhibition (independent of inhibitor) tetrahydrobiopterin ($\hbox {BH}_{4}$) is present. $\hbox {BH}_{4}$ is an essential co-factor for eNOS and plays an important role in normal endothelial cell functioning [25]. Despite of the mechanisms affecting the limited NO synthesis, NO can act positively and may lead to reduction of superoxide anion radical, which results in peroxynitrite production and inhibition of LDL oxidation. However, in conditions conducive to development of atherosclerosis (where the level of LDL is very high) the positive role of NO is irrelevant. In atherosclerosis, LDL oxidation occurs more often than inhibition of this process.

However, the analysis of clusters is too general in the context of disorders in prothrombotic states in atherosclerosis. Not very detailed information is included in biological descriptions of clusters $\hbox {c}_{6}$, $\hbox {c}_{7}$ and $\hbox {c}_{11}$ in Table 2. Cluster $\hbox {c}_{7}$ includes description of positive feedback of thrombin formation. Descriptions of clusters $\hbox {c}_{6}$ and $\hbox {c}_{11}$ pertain to endothelial damage and inflammation, which are caused by thrombin. For the purpose of obtaining additional information about disorders in prothrombotic states a more detailed analysis of particular t-invariants has been performed.

In the proposed model the extrinsic coagulation pathway consists of three pathways of positive feedback (Fig. 1). The more detailed analysis of t-invariants shows, that mentioned coagulation pathway consists of one or two or three pathways of positive feedback, which occur with different frequency:

- The biological description for only one t-invariant is associated with the extrinsic coagulation pathway which consists of three pathways of positive feedback. This t-invariant constitute only 0.05% of all the t-invariants.
- t-invariants for which the biological descriptions are associated with the extrinsic coagulation pathway which consists of one pathway (without the second pathway of positive feedback which normally leads to activation of factor VIII, and without the third pathway of positive feedback which normally leads to activation of factor XI). These t-invariants constitute 33.9% of all the t-invariants.
- t-invariants for which the biological descriptions are associated with the extrinsic coagulation pathway which consists of two pathways (without the third pathway of positive feedback which normally leads to activation of factor XI). These t-invariants constitute 33.7% of all the t-invariants.

The analysis of t-invariants allows to distinguish three different groups of t-invariants for which biological description is associated with the extrinsic pathway of coagulation. The first, the extrinsic pathway of coagulation which consists of three pathway of positive feedback is the rarest. The second, the extrinsic pathway of coagulation which consists of two pathways of positive feedback without one pathway (the third one) and the third, without two pathways (the second and the third one) occur with almost the same frequency about 34% of all the t-invariants.

Moreover, the biological descriptions of t-invariants can be divided into another two groups: the extrinsic pathway of coagulation which leads to reduction of fibrinogen to fibrin and the extrinsic pathway of coagulation without reduction of fibrinogen to fibrin:

- t-invariants for which the biological descriptions are associated with the extrinsic coagulation pathway consisting of three pathways which not leads to reduction of fibrinogen to fibrin. These t-invariants constitute 0.05% of all the t-invariants.
- t-invariants for which the biological descriptions are associated with the extrinsic coagulation pathway consisting of two pathways which not leads to reduction of fibrinogen to fibrin. These t-invariants constitute 26.7% of all the t-invariants.
- t-invariants for which the biological descriptions are associated with the extrinsic coagulation pathway consisting of one pathway which not leads to reduction of fibrinogen to fibrin. These t-invariants constitute 26.8% of all the t-invariants.
- t-invariants for which the biological descriptions are associated with the extrinsic coagulation pathway consisting of one pathway which leads to reduction of fibrinogen to fibrin. These t-invariants constitute 7.13% of all the t-invariants.
- t-invariants for which the biological descriptions are associated with the extrinsic coagulation pathway consisting of two pathways which leads to reduction of fibrinogen to fibrin. These t-invariants constitute 7.03% of all the t-invariants..
- t-invariants for which the biological descriptions are associated with reduction of fibrinogen to fibrin without the three pathways of positive feedback. These t-invariants constitute 3.25% of all the t-invariants.

Despite the lack of fibrin formation, atherosclerosis is still being promoted. It is because the proposed model includes other mechanisms engaged in thrombus formation, e.g., cigarette smoke induce thromboxane A2 and it leads to impaired prostacyclin production, which can block the coronary blood vessels and promote thrombus creation (direct influence on development of atherosclerosis).

Besides the obvious influence of thrombin on coagulation cascade, it is direct engaged in the development of atherosclerosis:

- t-invariants for which the biological descriptions are associated with the direct influence of thrombin on key processes engaged in the development of atherosclerosis constitute about 23% of all the t-invariants.. To be precise, thrombin is engaged in: proliferation (110 t-invariants which constitute 5.26%), inflammation (1 t-invariant which constitute 0.05%), endothelial dysfunction (5 t-invariants which constitute 0.24%), activation of platelets (364 t-invariants which constitute 17.42%).

## Conclusions

Cluster analysis distinguished main subprocesses and mechanisms, which are engaged in the development of atherosclerosis. Additionally, this analysis allowed to confirm some dependencies occurring between the modeled subprocesses and indicated how cigarette smoke is engaged in the atherosclerosis formation. The key processes are here: endothelial dysfunction, inflammation, NO synthesis with its dual role, cigarette smoking, the extrinsic pathway of coagulation and oxidative stress.

The analysis confirmed that oxidative stress (respiratory burst) and inflammation are key mechanisms that are associated with endothelial dysfunction. These processes result in increased secretion of ROS and free radicals, which are engaged in excessive LDL oxidation and decreasing NO bioavailability. The described processes lead to formation of plaque and in pathological conditions to plaque rupture and beginning of activation of clotting cascade, which can be additionally stimulated by disturbances in prothrombotic states.

This analysis showed how complex is the net of the interactions in the modeled process. Inflammation and oxidative stress are engaged in almost all of the paths which are described in Table 2. These phenomena are closely related with endothelial dysfunction and development of atherosclerosis. Additionally, cluster analysis showed how important influence on the development of atherosclerosis have processes closely related with cigarette smoking.

The more detailed analysis of particular t-invariants confirmed that coagulation process, which leads to thrombin formation, can act correctly even without two pathways of positive feedback. Some of t-invariants show that reduction of fibrinogen to fibrin via thrombin is not necessary for promotion of thrombus creation. In the proposed model, different mechanisms which can lead to creation of thrombus have been included. In some cases, the development of atherosclerosis can be stimulated by other processes, e.g., cigarette smoking stimulates creation of thrombus via activation of platelets, increased quantity of tissue factor, increasing of the viscosity of blood caused by catecholamines, blocking the coronary blood vessels caused by thromboxane A2 and by impaired prostacyclin production.

Using of the systems approach for selected aspects of tobacco-induced prothrombotic state allows for better understanding of atherosclerosis and dependencies between its important subprocesses.

### Electronic supplementary material

Below is the link to the electronic supplementary material.

Supplementary material 1 (pdf 68 KB)

## Appendix 1: List of non-trivial MCT sets

**Table 1:** List of non-trivial MCT sets

MCT set	Contained transitions	Biological interpretation
$m_{1}$	$t_{45}$, $t_{111}$, $t_{112}$, $t_{114}$, $t_{115}$, $t_{116}$, $t_{117}$, $t_{118}$, $t_{119}$	Thrombin leads to reduction of fibrinogen to fibrinopeptides and monomers. Thrombin also leads to activation of factor XIII to XIIIa. An activated factor XIII is engaged in polymerization of fibrin, which activates platelets and stimulates formation of thrombus
$m_{2}$	$t_{12}$, $t_{13}$, $t_{14}$, $t_{15}$, $t_{16}$, $t_{21}$, $t_{22}$	Partial NO synthesis, where eNOS co-factor ($\hbox {BH}_{4}$) and l-arginine do not occur
$m_{3}$	$t_{46}$, $t_{47}$, $t_{54}$, $t_{70}$, $t_{74}$, $t_{77}$	Cigarette smoke stimulates secretion of thromboxane A2 and it leads to impaired prostacyclin production, which can block the coronary blood vessels and directly promotes atherosclerosis. Cigarette smokes secrete also reactive oxygen species (ROS), free radicals and metals, which stimulate respiratory burst and formation of superoxide anion radical—this mechanism promotes LDL oxidation
$m_{4}$	$t_{56}$, $t_{57}$, $t_{58}$, $t_{59}$, $t_{60}$, $t_{61}$	Cigarette smoke secretes nicotine, which induces catecholamines. Catecholamines lead to decreased quantity of $\hbox {O}_{2}$ and increased quantity of CO, and additionally lead to activation of alpha receptors. This mechanisms lead to increased blood viscosity and promotes thrombus formation. This MCT set includes also polycyclic aromatic hydrocarbons, which induce chemokines (stimulation of inflammation in smokers)
$m_{5}$	$t_{81}$, $t_{82}$, $t_{83}$, $t_{84}$, $t_{85}$, $t_{102}$	Cigarette smoke leads to modification of lipid profile and also leads to decreased quantity of eNOS co-factor, which results in limited nitric oxide synthesis
$m_{6}$	$t_{39}$, $t_{42}$, $t_{43}$, $t_{49}$, $t_{50}$	Harmful acting of macrophages. Macrophages secrete cytokines and growth factor, which lead to proliferation. This amplified cells secrete collagen and matrix glycoproteins, which results in fibrous cap formation. Additionally, macrophages purpose is getting rid of damaged cell. In this case macrophages absorb oxidized LDL form (oxLDL)—this mechanism leads to creation of foam cells and then to destruction and release of necrotic core. Necrotic core and fibrous cap are engaged in plaque creation
$m_{7}$	$t_{109}$, $t_{110}$, $t_{124}$, $t_{125}$, $t_{126}$	Positive feedback of thrombin formation. Thrombin activates factor V to Va, which can create prothrombinase complex with factor Xa. Prothrombinase complex leads to transition of prothrombin to thrombin
$m_{8}$	$t_{87}$, $t_{90}$, $t_{92}$, $t_{93}$	Cigarette smoke leads to increased quantity of von Willebrand factor (which stimulates prothrombotic states), endothelin-1 (which can lead to endothelial dysfunction), and white blood cells count (which may favor the development of atherosclerosis)
$m_{9}$	$t_{94}$, $t_{120}$, $t_{121}$, $t_{139}$	TF leads to inhibition of plasminogen activator. This disorder in prothrombotic states promotes formation of thrombus and in consequence cardiovascular events
$m_{10}$	$t_{127}$, $t_{128}$, $t_{132}$, $t_{133}$	Positive feedback of thrombin formation. Activation of factor VIII to VIIIa, which can create tenase complex with factor IXa. Tenase complex activates factor X to Xa, which can lead to transition of prothrombin to thrombin
$m_{11}$	$t_{86}$, $t_{88}$, $t_{89}$	Cigarette smoke stimulates development of inflammation via increased quantity of macrophages, which secretes cytokines
$m_{12}$	$t_{104}$, $t_{105}$, $t_{107}$	Formation of TF-VIIa complex, which is necessary to factor Xa activation and leads to transition of prothrombin to thrombin
$m_{13}$	$t_{129}$, $t_{130}$, $t_{131}$	Activation of factor XI to IXa via thrombin
$m_{14}$	$t_{4}$, $t_{99}$	Endothelial damage caused by high blood pressure
$m_{15}$	$t_{5}$, $t_{100}$	Endothelial damage caused by toxins
$m_{16}$	$t_{6}$, $t_{101}$	Endothelial damage caused by other factors
$m_{17}$	$t_{10}$, $t_{11}$	NO synthesis is limited by the presence of ADMA (inhibitor of eNOS)
$m_{18}$	$t_{18}$, $t_{19}$	NO synthesis is limited by the presence of L-NMMA (inhibitor of l-arginine)
$m_{19}$	$t_{20}$, $t_{41}$	Inflammation response of endothelial damage: chemokines secretion and monocytes adhesion, which are engaged in diapedesis and transformation to macrophages
$m_{20}$	$t_{24}$, $t_{27}$	Correct NO synthesis: quantity of NO is sufficient to regulate blood pressure
$m_{21}$	$t_{28}$, $t_{141}$	Correct NO synthesis: quantity of NO is sufficient to inhibition of platelets aggregation
$m_{22}$	$t_{29}$, $t_{30}$	Incorrect NO synthesis: limited NO synthesis leads to decreased quantity of NO and increased quantity of superoxide anion radical, which promotes LDL oxidation
$m_{23}$	$t_{31}$, $t_{32}$	Positive role of NO: reduction of superoxide anion radical and NO to peroxynitrite, which inhibit LDL oxidation. This mechanism is observed in both cases: when NO synthesis is correct and when is limited
$m_{24}$	$t_{37}$, $t_{38}$	Typical inflammation response: monocytes adhesion and formation of monocytes and VCAM-1 complex lead to diapedesis and then to transformation to macrophages
$m_{25}$	$t_{40}$, $t_{48}$	Macrophages lead to secretion of cytokines and growth factors, which are engaged in proliferation
$m_{26}$	$t_{68}$, $t_{75}$	Harmful forms of LDL (oxLDL) lead to increased expression of ICAM-1, which is engaged in proliferation
$m_{27}$	$t_{79}$, $t_{80}$	Modifications of lipid profile (in smokers) lead to, inter alia, increased quantity of LDL and triglycerides. LDL can lead to endothelial damage
$m_{28}$	$t_{95}$, $t_{140}$	Correct NO synthesis can lead to inhibition of harmful mechanisms (for example, inhibition of macrophage colony stimulating factors (MCSF), which is indirectly engaged in proliferation)
$m_{29}$	$t_{103}$, $t_{113}$	Tissue damage leads to secretion of tissue factor and calcium ions. TF is correlated with disorders in prothrombotic states (in smokers) and disorders in clotting cascade
$m_{30}$	$t_{106}$, $t_{108}$	Activation of factor X to Xa via VIIa-TF complex
$m_{31}$	$t_{122}$, $t_{123}$	Activation of factor IX to IXa via VIIa-TF complex

The column “MCT set” contains names of MCT sets. The column “Contained transitions” includes names of transitions contained in a given MCT set. The column “Biological interpretation” includes biological description (functions and mechanisms) of MCT sets

## Appendix 2: List of t-clusters

**Table 2:** List of t-clusters

t-cluster	Biological meaning	t-cluster	Biological meaning
$c_{1}$	Limited NO synthesis caused by l-arginine inhibitor (L-NMMA). Despite of mechanisms affecting the limited synthesis, NO may act positively and may inhibit LDL oxidation	$c_{11}$	Endothelial dysfunction caused by various factors (e.g., high blood pressure, LDL, toxins, thrombin) leads to inhibition of eNOS, which results in limited NO synthesis. Despite of mechanisms affecting the limited synthesis, NO can act positively and may inhibit LDL oxidation (co-factor eNOS ($\hbox {BH}_{4}$) is present in mechanisms which limit NO synthesis via inhibition of eNOS)
$c_{2}$	Inflammatory response of endothelial damage: induction of chemokines, which stimulate adhesion of monocytes. In consequence monocytes can move out of the vessels wall (diapedesis) and they can transform into macrophages.	$c_{12}$	Endothelial dysfunction caused by all modeled factors (e.g., high LDL level (additionally stimulated by modification of lipid profile in smokers), high blood pressure, toxins, thrombin) stimulates inflammatory response. This cluster includes also correct NO synthesis, which can inhibit some of harmful mechanisms (e.g., inhibition of inflammation by inhibition of cytokines and VCAM-1)
$c_{3}$	Cigarette smoke leads to impairment of fibromuscular dysplasia (FMD): direct damage leads to tissue remodeling.	$c_{13}$	Endothelial dysfunction caused by toxins stimulates inflammatory response (induction of chemokines, which stimulate adhesion of monocytes)
$c_{4}$	Limited NO synthesis caused by inhibition of eNOS co-factor ($\hbox {BH}_{4}$). Despite of mechanisms affecting the limited synthesis, NO can act positively and may inhibit LDL oxidation	$c_{14}$	Endothelial dysfunction caused by other factors (e.g., high glucose level in diabetes) stimulates inflammatory response (induction of chemokines, which stimulate adhesion of monocytes)
$c_{5}$	Cigarette smoke leads to modification of lipid profile and decreased quantity of eNOS co-factor ($\hbox {BH}_{4}$), which results in limited NO synthesis.	$c_{15}$	Cigarette smoke leads to endothelial damage via impairment of fibromuscular dysplasia. Endothelial dysfunction induces inflammation response: induction of chemokines, which stimulate adhesion of monocytes
$c_{6}$	Thrombin leads to endothelial dysfunction and stimulates inflammation via induction of chemokines	$c_{16}$	Cigarette smoke leads to modification of lipid profile and leads to increased quantity of LDL and triglycerides, what results in endothelial dysfunction and inflammation
$c_{7}$	Positive feedback of thrombin formation. Thrombin leads to activation of factor V to Va. Activated factor V can form prothrombinase complex with Xa. This complex leads to transition of prothrombin to thrombin. Thrombin leads also to activation of VIII to VIIIa. Activated factors VIII and IXa can form tenase complex. This complex is engaged in activation of factor X to Xa, which results in transition of prothrombin to thrombin. Additionally, thrombin leads to activation of factor XI to XIa, which activates factor IX to IXa (activated factor IXa is a part of tenase complex)	$c_{17}$	Cigarette smoke leads to endothelial damage via impairment of fibromuscular dysplasia. Endothelial damage leads to inhibition of eNOS, which results in limited NO synthesis (co-factor eNOS ($\hbox {BH}_{4}$) is present in mechanisms which limit NO synthesis via inhibition of eNOS)
$c_{8}$	Almost all processes, which are included in the model, what shows how dynamic and complex is atherosclerosis	$c_{18}$	Cigarette smoke leads to modification of lipid profile and increased quantity of LDL, which results in endothelial damage. Dysfunction of endothelium leads to inhibition of eNOS and limits NO synthesis (co-factor eNOS ($\hbox {BH}_{4}$) is present in mechanisms which limit NO synthesis via inhibition of eNOS)
$c_{9}$	Limited NO synthesis caused by eNOS inhibitor (ADMA). Despite of mechanisms affecting the limited synthesis, NO can act positively and may inhibit LDL oxidation [co-factor eNOS ($\hbox {BH}_{4}$) is present in mechanisms which limit NO synthesis via inhibition of eNOS]	$c_{19}$	Cigarette smoke stimulates development of inflammatory area via stimulation of macrophages. Macrophages secret cytokines, which are engaged in respiratory burst and promotion of LDL oxidation, but also are engaged in proliferation together with growth factor. These amplified cells secrete collagen and matrix glycoproteins, what results in fibrous cap formation and promotion of atherosclerosis. Proliferation can be inhibited by NO, when NO synthesis is correct
$c_{10}$	Endothelial dysfunction influenced by high blood pressure stimulates inflammatory response, what means, that chemokines stimulate adhesion of monocytes		

The column “t-cluster” contains the names of t-clusters, while the column “Biological meaning” includes their biological interpretations
