# Supplementary material for: Selected Aspects of Tobacco-Induced Prothrombotic State, Inflammation and Oxidative Stress: Modeled and Analyzed Using Petri Nets
Source: Interdiscip Sci. 2018 Dec 24;11(3):373–86. doi: 10.1007/s12539-018-0310-7 (PMC6660494; doi:10.1007/s12539-018-0310-7)
Supplement: Supplementary file 1 — Supplementary material 1 (pdf 68 KB) [file 12539_2018_310_MOESM1_ESM.pdf]

Noname manuscript No.
(will be inserted by the editor)

Selected aspects of tobacco-induced prothrombotic state, inflammation and oxidative stress – modeled and analyzed using Petri nets

Kaja Gutowska · Dorota Formanowicz ·
Piotr Formanowicz

Received: date / Accepted: date

Table S1: List of places.

No.	Biological meaning	No.	Biological meaning
p_0	healthy endothelium	p_{53}	oxidized LDL (oxLDL)
p_1	damaged endothelium	p_{54}	reactive oxygen species (ROS)
p_2	low-density lipoprotein (LDL)	p_{55}	free radicals
p_3	high blood pressure	p_{56}	cadmium
p_4	toxins	p_{57}	aldehydes
p_5	other factors	p_{58}	metals
p_6	endothelial nitric oxide synthase (eNOS)	p_{59}	thrombus
p_7	asymmetric dimethylarginine (ADMA)	p_{60}	platelets
p_8	inducible nitric oxide synthase (iNOS)	p_{61}	lipid profile
p_9	neuronal nitric oxide synthase (nNOS)	p_{62}	triglyceride
p_{10}	NADPH	p_{63}	high-density lipoprotein (HDL)
p_{11}	L-arginine	p_{64}	fibromuscular dysplasia (FMD)
p_{12}	dioxygen (O_2)	p_{65}	apolipoprotein A1
p_{13}	NG-monomethyl L-arginine (L-NMMA)	p_{66}	selenium
p_{14}	citrulline	p_{67}	less quantity of tetrahydrobiopterin (BH_4)
p_{15}	NADP	p_{68}	lymphocytes

K. Gutowska
Institute of Computing Science, Poznan University of Technology, Piotrowo 2, 60-965 Poznan, Poland.
E-mail: Kaja.Gutowska@cs.put.poznan.pl

D. Formanowicz
Department of Clinical Biochemistry and Laboratory Medicine, Poznan University of Medical Sciences, Rokietnicka 8, 60-806 Poznan, Poland.
E-mail: doforman@ump.edu.pl

P. Formanowicz
Institute of Computing Science, Poznan University of Technology, Piotrowo 2, 60-965 Poznan, Poland.
Institute of Bioorganic Chemistry, Polish Academy of Sciences, Noskowskiego 12/14, 61-704 Poznan, Poland.
E-mail: Piotr.Formanowicz@cs.put.poznan.pl

No.	Biological meaning	No.	Biological meaning
p_{16}	insufficient quantity of nitric oxide (damaged endothelium)	p_{69}	neutrophils
p_{17}	sufficient quantity of nitric oxide (healthy endothelium)	p_{70}	macrophages
p_{18}	nitric oxide (NO)	p_{71}	endothelin-1
p_{19}	blood pressure	p_{72}	plasminogen activator inhibitor-1 (PAI-1)
p_{20}	platelet aggregation	p_{73}	von Willebrand factor
p_{21}	amplified cell caused by proliferation vascular smooth muscle cells (VSMC)	p_{74}	decreased quantity of NO
p_{22}	peroxinitrite	p_{75}	plasminogen activator
p_{23}	superoxide anion radical	p_{76}	other mechanisms
p_{24}	intercellular adhesion molecule-1 (ICAM-1)	p_{77}	BH_4 co-factor of eNOS
p_{25}	adhesion molecules	p_{78}	damaged tissue
p_{26}	inactive monocyte	p_{79}	VII
p_{27}	active monocyte	p_{80}	VIIa
p_{28}	vascular cell adhesion protein-1 (VCAM-1)	p_{81}	calcium ion
p_{29}	VCAM-monocyte complex	p_{82}	TF-VIIa complex
p_{30}	foam cell	p_{83}	X
p_{31}	necrosis core	p_{84}	Xa
p_{32}	plaque	p_{85}	prothrombin
p_{33}	TF	p_{86}	thrombin
p_{34}	atherosclerosis	p_{87}	fibrinogen
p_{35}	growth factor	p_{88}	monomers
p_{36}	cytokines	p_{89}	fibrinopeptides
p_{37}	matrix glycoproteins	p_{90}	XIIIa
p_{38}	collagen	p_{91}	XIII
p_{39}	fibrous cap	p_{92}	platelet factor 4 (PF4)
p_{40}	white blood cells	p_{93}	loose fibrin
p_{41}	cigarette smoke	p_{94}	fibrin
p_{42}	thromboxane A2	p_{95}	IX
p_{43}	decreased quantity of prostacyclin caused by impaired production via smoking	p_{96}	IXa
p_{44}	nicotine	p_{97}	V
p_{45}	polycyclic aromatic hydrocarbon	p_{98}	Va
p_{46}	chemokines	p_{99}	prothrombinase complex
p_{47}	matrix metalloproteinases (MMP)	p_{100}	VIII
p_{48}	catecholamines	p_{101}	VIIIa
p_{49}	carbon monoxide (CO)	p_{102}	XI
p_{50}	alpha receptors	p_{103}	XIa
p_{51}	highly viscous blood	p_{104}	tenase
p_{52}	impairment of FMD	p_{105}	prothrombotic states

Table S2: List of transitions.

No.	Biological meaning	No.	Biological meaning
t_0	damage caused by LDL	t_{73}	stimulation of prothrombotic and procoagulative states caused by increase of platelets
t_1	secretion by damaged endothelial	t_{74}	stimulation of oxidation caused by metals
t_2	auxiliary transition 1	t_{75}	stimulation caused by ICAM-1
t_3	auxiliary transition 2	t_{76}	stimulation caused by adhesion molecules

No.	Biological meaning	No.	Biological meaning
t_4	auxiliary transition 3	t_{77}	stimulation of respiratory burst caused by free radicals
t_5	auxiliary transition 4	t_{78}	modification caused by cigarette smoke
t_6	auxiliary transition 5	t_{79}	increase caused by modification of lipid profile
t_7	expression by damaged endothelial	t_{80}	auxiliary transition 25
t_8	inhibition of eNOS caused by damaged endothelium	t_{81}	decrease caused by modification of lipid profile
t_9	secretion by healthy endothelium	t_{82}	remodeling tissue caused by FMD
t_{10}	inhibition of eNOS caused by ADMA	t_{83}	auxiliary transition 26
t_{11}	auxiliary transition 6	t_{84}	auxiliary transition 27
t_{12}	auxiliary transition 7	t_{85}	auxiliary transition 28
t_{13}	auxiliary transition 8	t_{86}	increase caused by development of inflammatory environment
t_{14}	synthesis of NO	t_{87}	increase caused by cigarette smoke
t_{15}	auxiliary transition 9	t_{88}	auxiliary transition 29
t_{16}	auxiliary transition 10	t_{89}	auxiliary transition 30
t_{17}	auxiliary transition 11	t_{90}	creation caused by white blood cells
t_{18}	inhibition of L-arginine caused by L-NMMA	t_{91}	creation caused by LDL
t_{19}	auxiliary transition 12	t_{92}	damage caused by endothelin-1
t_{20}	auxiliary transition 13	t_{93}	stimulation of prothrombotic states caused by von Willebrand factor
t_{21}	auxiliary transition 14	t_{94}	stimulation of prothrombotic states caused by plasminogen activator
t_{22}	auxiliary transition 15	t_{95}	auxiliary transition 31
t_{23}	high quantity of NO	t_{96}	increasing the affinity of eNOS to L-arginine
t_{24}	regulation caused by NO (healthy endothelium)	t_{97}	inhibition of BH_4 co-factor
t_{25}	inhibition of amplified cell caused by NO (healthy endothelium)	t_{98}	auxiliary transition 32
t_{26}	inhibition of molecules adhesion	t_{99}	damage caused by high blood pressure
t_{27}	auxiliary transition 16	t_{100}	damage caused by toxins
t_{28}	auxiliary transition 17	t_{101}	damage caused by other factor
t_{29}	low quantity of NO	t_{102}	inhibition of BH_4 caused by smoking
t_{30}	high quantity of superoxide anion radical	t_{103}	secretion of TF
t_{31}	reduction	t_{104}	activation of VII
t_{32}	inhibition of oxLDL	t_{105}	creation of TF-VIIa complex
t_{33}	oxidation	t_{106}	activation of X
t_{34}	auxiliary transition 18	t_{107}	auxiliary transition 33
t_{35}	monocyte activation by chemokines	t_{108}	auxiliary transition 34
t_{36}	auxiliary transition 19	t_{109}	activation of II
t_{37}	adhesion of monocytes	t_{110}	auxiliary transition 35
t_{38}	transformation	t_{111}	reduction of fibrinogen
t_{39}	uptake	t_{112}	activation of XII
t_{40}	secretion by macrophage	t_{113}	auxiliary transition 36
t_{41}	auxiliary transition 20	t_{114}	auxiliary transition 37
t_{42}	destruction of foam cell	t_{115}	auxiliary transition 38
t_{43}	create plaque caused by necrosis core and fibrous cap	t_{116}	polymerization
t_{44}	plaque rapture	t_{117}	auxiliary transition 39
t_{45}	activate of blood platelets	t_{118}	fibrin stabilization
t_{46}	block the coronary blood vessels	t_{119}	auxiliary transition 40
t_{47}	auxiliary transition 21	t_{120}	inhibition of plasminogen activator

No.	Biological meaning	No.	Biological meaning
t_{48}	proliferation caused by growth factor and cytokines	t_{121}	increasing quantity of PAI-1
t_{49}	secretion by amplified cells	t_{122}	auxiliary transition 41
t_{50}	creation of fibrous cap	t_{123}	activation of IX
t_{51}	auxiliary transition 22	t_{124}	activation of V
t_{52}	respiratory burst	t_{125}	auxiliary transition 42
t_{53}	auxiliary transition 23	t_{126}	creation of prothrombinase complex
t_{54}	secretion by cigarette smoke	t_{127}	auxiliary transition 43
t_{55}	activation caused by nicotine and polycyclic aromatic hydrocarbon	t_{128}	activation VIII
t_{56}	induction caused by nicotine	t_{129}	activation IXa via XIa
t_{57}	induction caused by polycyclic aromatic hydrocarbon	t_{130}	activation of XI
t_{58}	decreasing quantity of O_2 and increasing quantity of CO	t_{131}	auxiliary transition 44
t_{59}	activation caused by catecholamines	t_{132}	creation of tenase complex
t_{60}	increasing of the viscosity of blood	t_{133}	activation of X caused by tenase complex
t_{61}	stimulation caused by highly viscous blood	t_{134}	endothelial dysfunctions caused by trombin
t_{62}	stimulation caused by matrix metalloproteinases	t_{135}	increase expression of chemokines by thrombin
t_{63}	direct damage caused by cigarette smoke	t_{136}	stimulation caused by thrombin
t_{64}	remodeling of tissue caused by impairment of FMD	t_{137}	activation of platelets caused by thrombin
t_{65}	damage caused by impairment of FMD	t_{138}	auxiliary transition 45
t_{66}	auxiliary transition 24	t_{139}	auxiliary transition 46
t_{67}	expression of molecules adhesion caused by oxLDL	t_{140}	inhibition of other mechanisms
t_{68}	expression caused by oxLDL	t_{141}	inhibition of platelet aggregation
t_{69}	expression caused by cadmium	t_{142}	inhibition of cytokines
t_{70}	increase caused by aldehydes	t_{143}	auxiliary transition 47
t_{71}	role in endothelial cell death	t_{144}	inhibition of VCAM-1
t_{72}	increase and activation caused by cigarette smoke		
